# Correction: Education for non-citizen children in Malaysia during the COVID-19 pandemic: A qualitative study

**DOI:** 10.1371/journal.pone.0314491

**Published:** 2024-11-20

**Authors:** Tharani Loganathan, Zhie X. Chan, Fikri Hassan, Watinee Kunpeuk, Rapeepong Suphanchaimat, Huso Yi, Hazreen Abdul Majid

The S1 File uploaded is incorrect. The file should be Definition of Terms which is omitted from the list of Supporting Information. Meanwhile, the Interview guide should be S2 File. The correct S1 and S2 Files can be viewed below.

## Supporting information

S1 File (DOCX)

S2 File (PDF)
