# Supplementary material for: Correction: Education for non-citizen children in Malaysia during the COVID-19 pandemic: A qualitative study
Source: PLoS One. 2024 Nov 20;19(11):e0314491. doi: 10.1371/journal.pone.0314491 (PMC11578443; doi:10.1371/journal.pone.0314491)
Supplement: S1 File — (DOCX) [file pone.0314491.s001.docx]

**Definition of Terms**

This study focuses on refugee and asylum seeker, migrant, stateless and undocumented children in Malaysia. International students and children of expatriates were excluded from this study.

Refugees are those forcibly displaced from their country of nationality or usual residence and are unable or unwilling to return due to a well-founded fear of persecution [3, 26]. Refugees are recognised and protected by international law. Asylum-seekers are individuals seeking international protection, but whose refugee status has yet to be officially determined.

Migrant children refer to children of low-skilled migrant workers. Since immigration laws and employment contracts prohibit migrant workers from bringing dependents or from having children in Malaysia, migrant children are likely to have unregistered births and to be undocumented.

According to the 1954 Convention relating to the Status of Stateless Persons, stateless persons are not considered as nationals by any State under the operation of its law and this includes persons with undetermined nationality [4].

The term undocumented migrant refers to anyone residing in the country without legal documentation, including people who entered the country without valid passports or permits, and children without birth certificates.
