# Supplementary material for: Correction: Education for non-citizen children in Malaysia during the COVID-19 pandemic: A qualitative study
Source: PLoS One. 2024 Nov 20;19(11):e0314491. doi: 10.1371/journal.pone.0314491 (PMC11578443; doi:10.1371/journal.pone.0314491)
Supplement: S2 File — (PDF) [file pone.0314491.s002.pdf]

Table of Contents

A. SCHOOL HEAD TEACHER, TEACHERS, EDUCATORS 2

- TOPICS 2
- INTRODUCTORY QUESTIONS 2
- OPEN QUESTIONS 2

B. PARENTS/FAMILY MEMBERS, MIGRANT REPRESENTATIVES, NGOS 5

- TOPICS 5
- INTRODUCTORY QUESTIONS 5
- OPEN QUESTIONS 5

C. POLICY MAKERS/ANALYSTS AND OTHER HIGH-LEVEL STAKEHOLDERS 7

- TOPICS 7
- INTRODUCTORY QUESTIONS 7
- OPEN QUESTIONS 7

A. School Head Teacher, Teachers, Educators

Interview topics and questions that form the broad framework of discussion: education policies for migrants in Malaysia

Topics

- Knowledge of education policy and services on migrants* in Malaysia
- Experience with migrant* access to education in Malaysia
- Perceptions or experience of barriers to migrant* access to education in Malaysia
- Experiences of education providers – challenges in implementations
- Knowledge and experience of health services/policies offered to migrants*
- Suggestion for improvement in education policy or services for migrants*

*Non-citizen students: children, adolescents, adults

Introductory questions

Please tell us about –

1. Your organisation:
   Prompt:
   (a) Core mandates
   (b) Scope and breath of services
   (c) Curriculum/syllabus/modules – instrument used or compliance with any standards
   (d) Funders if relevant (SKIP IF NECESSARY)
   (e) Resources (voluntary, paid roles)

2. Your role in your organisation:
   Prompt:
   (a) Duration served
   (b) Responsibilities
   (c) Past experiences in the job

3. Your daily job with regards to migrant students:
   Prompt:
   (a) Do you have many migrants coming to your facility every day?
   (b) How many migrant children are enrolled in your school? (age, gender, country of origin)
   (c) Outcomes from their participation in your service (e.g. graduation, further education, work opportunities, etc.)

Open questions

Please tell us about –

4. The education policies pertaining migrant students in Malaysia
   Prompt:
   (a) The act and legislation if known
   (b) The governing body for the centre/school
   (c) What your organisation can do and cannot do
   (d) Grey spaces in the operation of education services, if any (potentially sensitive, SKIP OF NECESSARY)

Note: The introductory questions are targeted to interviewees according to their backgrounds

5. The process of migrant students enrolling in an education programme (in public schools, learning centres, government aided schools, private institutions, and so on)?
   Prompt:
   (a) The admission's standard operating procedure (SOP) for a non-citizen/migrant be enrolled into the school or education programme
   (b) Fees

6. The differences of local and migrant students in terms of
   Prompt:
   (a) Fees
   (b) Learning modules (syllabus)
   (c) Primary language /aptitude in local language
   (d) Cultural differences (fit in to school environment, discipline etc.)
   (e) Performance in lessons
   (f) Provider treatment differences (preferential)

7. The barriers to migrant access to education in Malaysia
   Prompt:
   (a) Common barriers seen in migrant students, when it comes to accessing education in Malaysia
   (b) Legal status/documentation
   (c) Language barrier
   (d) Cost of education
   (e) Known barriers in access be between male/female/others migrant students
   (f) Others

8. The challenges in the delivery of the education programme
   Prompt:
   (a) Teaching migrant children
   (b) Are there language barriers? Are lessons conducted in migrant languages/using instructors familiar with migrant languages/culture
   (c) Are there cultural barriers? Particularly for girls/boys etc
   (d) Poor foundational background in reading/writing etc
   (e) Parental support or background?
   (f) Any other barriers/challenges

9. What has worked well in the delivery of services? Contributor/facilitator of success stories
   Prompt:
   (a) Education programme
   (b) School health programme: dental, immunisation, health education, environmental health, etc.

10. Has there been an evaluation on migrant students experience the services (education programme)? E.g. as part of the funder's requirement.
    Prompt:
    (a) Specific to the programme offered
    (b) At a broad level (e.g. national)
    (c) The evidence available/published (data and statistics)

11. Your suggestion for improvement in education policy or services for migrants
    Prompt:
    (a) Policy that needs to be reformed/amended
    (b) Awareness of any on-going reforms
    (c) Area that needs to be addressed to improve migrants' access to education

12. What are the impacts, issues and challenges arising of COVID-19 pandemic on education for migrants?

    Prompt:
    (a) Impact, issues and challenges faced (school closure, ability to continue education at home, resources such as the internet access, devices, books)
    (b) Arrangement/process that has worked well and not so well
    (c) The appropriate steps to take to address these impacts, issues, and challenges
    (d) Other suggestions for improvements

13. Your final thoughts and comments (before I end this interview)

    Prompt:
    (a) Importance on migrant-education
    (b) Foreseeable future impact to the children/nation
    (c) Sustainability of education programmes
    (d) Role of the government (note the shift of power in early 2020)

B. Parents/family members, Migrant representatives, NGOs

Interview topics and questions that form the broad framework of discussion: education policies for migrants in Malaysia

Topics

- Knowledge of education policy and services on migrants* in Malaysia
- Experience with migrant* access to education in Malaysia
- Perceptions or experience of barriers to migrant* access to education in Malaysia
- Experiences of education providers – challenges in implementations
- Knowledge and experience of health services/policies offered to migrants*
- Suggestion for improvement in education policy or services for migrants*

*Non-citizen students: children, adolescents, adults

Introductory questions

For parents/family members:

1. Please tell me about yourself
   Prompt:
   (a) Occupation(s) (formal vs informal)
   (b) Contract type (contracted/not contracted, daily vs monthly wage)
   (c) Some of the migration history (country of origin and years been in Malaysia)
   (d) Community affiliation/associated with (localisation and integration)

2. Please tell me about your family
   Prompt:
   (a) Family members
   (b) Number of children in schools (Female/Male)
   (c) Having to sustain him/herself by working or not

3. How do you support your family?
   Prompt:
   (a) Estimated income for the family
   (b) Estimated monthly expenses of the family

For representatives of migrant workers communities:

1. Please tell me about your community/organisation
   Prompt:
   (a) Role
   (b) Communities or nationalities the organisation represents
   (c) Types of migrants: age, sex, occupation, documented/undocumented, migrant workers/ refugees/ stateless people

Open questions

Please tell us about –

1. Your experience with schools and education services for migrant students in Malaysia
   Prompt:
   (a) Awareness of education options available for his/her children (elaborate)
   (b) Options taken (or will be chosen) from the following: public schools, learning centres, government aided schools, private institutions, and so on.
   (c) Provide reasons/rationale

Note: The introductory questions are targeted to interviewees according to their backgrounds

2. The barriers to migrant access to education in Malaysia
   Prompt:
   (a) Aware of requirements for enrolment (legal identity and documents)
   (b) Fees and affordability
   (c) Language
   (d) Cultural
   (e) Known barriers in access be between male/female/others migrant students
   (f) Others

3. Do you feel that the schools fulfil your children's education/learning needs? Explain.
   Prompt
   (a) Literacy/syllabus
   (b) Skills training
   (c) Ability to get a better job in Malaysia/elsewhere
   (d) Further education opportunities in Malaysia/elsewhere
   (e) Others

4. How has your child's identity as a migrant affected his/her ability to access education?
   Prompt
   (a) Felt discriminated against because of being a migrant in the school setting
   (b) Elaborate on possible reasons: e.g. gender/migrant status/social economical/language/culture/education level

5. What has worked well in the delivery of services? Contributor/facilitator of success stories
   Prompt:
   (a) Education programme
   (b) School health programme: dental, immunisation, health education, environmental health, etc.

6. What are the impacts, issues and challenges arising of COVID-19 pandemic on education for migrants?
   Prompt:
   (a) Impact, issues and challenges faced (school closure, ability to continue education at home, resources such as the internet access, devices, books)
   (b) Arrangement/process that has worked well and not so well
   (c) The appropriate steps to take to address these impacts, issues, and challenges
   (d) Other suggestions for improvements

7. Your suggestion for improvement in education policy or services for migrants
   Prompt:
   (a) Any policy that needs to be reformed/amended
   (b) Awareness of any on-going reforms
   (c) What needs to be addressed to improve migrants' access to education

8. Your final thoughts and comments (before I end this interview)
   Prompt:
   (a) Importance on migrant-education
   (b) Foreseeable future impact to the students
   (c) Sustainability of (a) and (b)
   (d) Role of the government (note the shift of power in early 2020)

C. Policy makers/analysts and other high-level stakeholders

Interview topics and questions that form the broad framework of discussion: education policies for migrants in Malaysia

Topics

- Knowledge of education policy and services on migrants* in Malaysia
- Experience with migrant* access to education in Malaysia
- Perceptions or experience of barriers to migrant* access to education in Malaysia
- Experiences of education providers – challenges in implementations
- Knowledge and experience of health services/policies offered to migrants*
- Suggestion for improvement in education policy or services for migrants*

*Non-citizen students: children, adolescents, adults

Introductory questions

1. Your organisation
   Prompt:
   (a) The mandates/duties of your organisation
   (b) Scope and breadth: Its work involving the topic of education
   (c) Other divisions/entities/ministries/special groups involved – efforts in alignment/means of working together

2. Your role in your organisation
   Prompt:
   (a) Occupation: role at the ministry/think tank
   (b) Professional experience involving the topic of education and in what capacity (responsibilities)

Open questions

Please tell us about –

3. The education policies pertaining to migrant students in Malaysia
   Prompt:
   (a) The act and legislation if known
   (b) The governing body for the centre/school
   (c) Adequacy of coverage for various groups (refugees/asylum seekers, stateless children, children of migrant workers etc)

4. The process/requirements for enrolling migrant students in an education programme (in public schools, learning centres, government aided schools, private institutions, and so on)?
   Prompt:
   (a) The admission's standard operating procedure (SOP) for a non-citizen/migrant be enrolled into the school or education programme
   (b) Documentation – BC, passport
   (c) Fees

5. Awareness of barriers to migrant access to education in Malaysia
   Prompt:
   (a) Requirements for enrolment (legal identity and documents)
   (b) Fees and affordability
   (c) Language
   (d) Cultural

Note: The introductory questions are targeted to interviewees according to their backgrounds

(e) Known differences in access for male/female/others migrant students
(f) Others

6. The differences of local and migrant students in terms of
   Prompt:
   (a) Fees
   (b) Learning modules (syllabus)
   (c) Primary language /aptitude in local language
   (d) Cultural differences (fit in to school environment, discipline etc.)
   (e) Performance in lessons
   (f) Provider treatment differences (preferential)

7. The challenges in the delivery of the education programme
   Prompt:
   (a) Teaching migrant children
   (b) Are there language barriers? Are lessons conducted in migrant languages/using instructors familiar with migrant languages/culture
   (c) Are there cultural barriers? Particularly for girls/boys etc
   (d) Poor foundational background in reading/writing etc
   (e) Parental support or background?
   (f) Any other barriers/challenges

8. Has there been an evaluation on migrant students experience the services (education programme)? E.g. as part of the funder's requirement.
   Prompt:
   (a) Specific to the programme offered
   (b) At a broad level (e.g. national)
   (c) The evidence available/published (data and statistics)

9. What has worked well in the delivery of services? Contributor/facilitator of success stories
   Prompt:
   (a) Education programme
   (b) Special programmes (e.g., mobile registration programme), special exemptions (I,e., in terms of documentation)
   (c) School health programme: dental, immunisation, health education, environmental health, etc.

10. Your suggestion for improvement in education policy or services for migrants
   Prompt:
   (a) Any policy that needs to be reformed/amended
   (b) Awareness of any on-going reforms
   (c) What needs to be addressed to improve migrants' access to education

11. What are the impacts, issues and challenges arising of COVID-19 pandemic on education for migrants?
   Prompt:
   (a) Impact, issues and challenges faced (school closure, ability to continue education at home, resources such as the internet access, devices, books)
   (b) Arrangement/process that has worked well and not so well
   (c) The appropriate steps to take to address these impacts, issues, and challenges
   (d) Other suggestions for improvements

12. Your final thoughts and comments (before I end this interview)
   Prompt:
   (a) Importance on migrant-education
   (b) Foreseeable future impact to the students

(c) Sustainability of education programmes
(d) Role of the government (note the shift of power in early 2020)
